# The Path to Sustainable Healthcare: Implementing Care Transition Teams to Mitigate Hospital Readmissions and Improve Patient Outcomes

**DOI:** 10.7759/cureus.39022

**Published:** 2023-05-15

**Authors:** Rajmohan Rammohan, Melvin Joy, Sai Greeshma Magam, Dilman Natt, Achal Patel, Olawale Akande, Robert M Yost, Susan Bunting, Prachi Anand, Paul Mustacchia

**Affiliations:** 1 Gastroenterology, Nassau University Medical Center, East Meadow, USA; 2 Internal Medicine, Nassau University Medical Center, East Meadow, USA; 3 Internal Medicine, Nassau University Medical Center, East Meadow , USA; 4 Rheumatology, Nassau University Medical Center, East Meadow, USA; 5 Gastroenterology and Hepatology, Nassau University Medical Center, East Meadow, USA

**Keywords:** health care transition, care transition, 30-day readmission rate, hospital readmission rate, social determinants of health (sdoh), health equity

## Abstract

Introduction

Hospital readmissions within 30 days suggest care quality issues and increased mortality risks. They result from ineffective initial treatment, poor discharge planning, and inadequate post-acute care. These high readmission rates harm patient outcomes and financially strain healthcare institutions, inviting penalties and discouraging potential patients. Enhancing inpatient care, care transitions, and case management is crucial to lowering readmissions. Our research underscores the role of care transition teams in reducing readmissions and financial stress in hospitals. By consistently applying transition strategies and focusing on high-quality care, we can improve patient outcomes and ensure hospital success in the long run.

Methods

This two-phase study investigated readmission rates and risk factors in a community hospital from May 2017 to November 2022. Phase 1 determined a baseline readmission rate and identified individual risk factors using logistic regression. In phase 2, a care transition team addressed these factors by providing post-discharge patient support through phone calls and assessing social determinants of health (SDOH). Readmission data from the intervention period was compared to baseline data using statistical tests. Data, including demographics, medical conditions, and comorbidities, were collected via electronic medical records and the International Classification of Diseases (ICD-10 codes). The study focused on patients aged 20-80 with readmissions within 30 days. Exclusions were made to minimize confounding effects from unmeasured comorbidities and ensure an accurate representation of factors affecting readmissions.

Results

In the study's initial phase, 74,153 patients participated, with an 18% mean readmission rate. Women accounted for 46% of readmissions, and the white population had the highest rate (49%). The 40-59 age group showed a higher readmission rate than other age groups, and certain health factors were identified as risk factors for 30-day readmission. In the subsequent phase, a care transition team intervened with high-risk groups using an SDOH questionnaire. They contacted 432 patients, resulting in a reduced overall readmission rate of 9%. The 60-79 age group and the Hispanic population experienced higher readmission rates, and the previously identified health factors remained significant risk factors.

Conclusion

This study emphasizes the crucial role of care transition teams in reducing hospital readmission rates and easing the financial strain on healthcare institutions. By identifying and addressing individual risk factors, the care transition team effectively lowered the overall readmission rate from 18% to 9%. Continually implementing transition strategies and prioritizing high-quality care focused on minimizing readmissions are essential for improving patient outcomes and long-term hospital success. Healthcare providers should consider utilizing care transition teams and social determinants of health assessments to better understand and manage risk factors and tailor post-discharge support for patients at higher risk of readmission.

## Introduction

Hospital readmissions occur when a patient is readmitted to an acute care hospital within a specified time frame, typically 30 days after discharge [1]. This metric assesses the quality of care provided to hospitalized patients, as those readmitted within this time frame face increased short- and long-term mortality risks [2]. Factors contributing to readmissions include inadequate treatment during the initial hospital stay, poor discharge planning, and a lack of coordination with post-acute care providers [3].

Readmissions adversely affect patient outcomes and impose a significant financial burden on healthcare institutions [3]. Elevated readmission rates can result in severe financial penalties and deter potential patients from utilizing a hospital's services [2]. Hospitals must prioritize optimizing inpatient care, care transitions, and case management to mitigate these issues [2]. Consistently implementing care transition strategies for patients is essential for reducing readmission rates [3]. Hospitals must deliver high-quality care and services focused on minimizing readmission rates while maintaining long-term financial sustainability [4].

Our study aims to emphasize the role of a care transition team in reducing readmission incidences and alleviating the financial strain experienced by healthcare institutions. By optimizing inpatient care, care transitions, and case management, hospitals can achieve a decline in mortality rates and overall improved healthcare outcomes [2]. Early reduction of readmissions can be facilitated by consistently applying care transition strategies for all patients [3]. Providing high-quality care focused on reducing readmissions is crucial for the long-term success of hospitals [4].

## Materials and methods

Study design

This two-phase study examined patient readmission rates and risk factors. The initial phase established a baseline readmission rate by gathering 30-day readmission data from a community hospital between May 2017 and June 2022. Logistic regression was applied to identify individual risk factors, and the odds ratio (OR) was used to confirm the risk factors.

In the second phase, a care transition team was formed in September 2022 to address these risk factors. Patients discharged from the Department of Medicine in September and October of 2022 received phone calls to facilitate smooth transitions. The Social Determinants of Health (SDOH) Questionnaire assessed healthcare access, socioeconomic status, education, housing, transportation, and physical environment quality upon discharge. Additionally, the study collected data on discharge medication prescriptions, follow-up appointments, and primary doctor appointments.

Readmission data from October to November 2022 was compared to the baseline data using the Chi-square test for categorical variables and the T-test for continuous variables. Before analysis, the baseline characteristics of each group were matched to ensure a fair comparison.

Data source

Data were collected using the Sunrise Electronic Medical Record software (Allscripts, Chicago, IL). The data collected included detailed information such as age, race, gender, 30-day mortality, length of stay, medication, alcohol abuse, liver disorders, hypertension, drug abuse, hyperlipidemia, and comorbidities. This information was obtained using International Classification of Diseases (ICD) 10 codes, including K92.2, K29.71, I86.4, I10, I63.9, D64.9, K70.3, K74.4, K74.5, K25.9, E08, E78.5, and K21.9.

Inclusion and exclusion criteria

The study selected patients aged 20-80 who experienced readmission within 30 days of discharge at Nassau University Medical Center in East Meadow, New York, from May 2017 to November 2022. However, patients with incomplete baseline comorbidity data and over three admissions due to alcohol and drug abuse were excluded. This exclusion aimed to prevent potential confounding effects from unmeasured or unknown comorbidities that could impact patient outcomes, ensuring a more accurate representation of the factors affecting readmissions.

Analysis

Data analysis was conducted using the Statistical Package for Social Sciences (SPSS) for Windows (IBM SPSS Statistics, Armonk, NY), RStudio software (RStudio, PBC, Boston, MA), and Joinpoint version 4.9.1.0, desktop version (National Cancer Institute, Bethesda, MD). Categorical data were represented by patient numbers (n).

## Results

In the initial phase of the study, 74,153 patients participated. The mean readmission rate was 13,347 (18%), with 6,140 (46%) being women. The white population accounted for 6539 (49%) participants, while African Americans made up 2670 (20%), Asians 667 (5%), Hispanics 2136 (16%), and other races 1335 (10%), as displayed in Table 1.

**Table 1:** Baseline readmission patient demographic Values are presented as numbers (n). Other races: Middle Eastern, South Asian.

	Total cases n = 13,347	Demographic in percentage %
Sex		
Female	6140	46%
Male	7207	54%
Race		
African American	2670	20%
White	6539	49%
Asians	667	5%
Hispanic	2136	16%
Others	1335	10%
Age		
20–39	4538	34%
40–59	5205	39%
60–79	3604	27%

The age group of 40-59 years had a higher readmission rate compared to the 20-39 and 60-79 age groups (39% vs. 34% & 27%, p<0.01 [0.70-0.81]). The white population experienced a higher readmission rate than other races (49% vs. 20% vs. 5% vs. 16% vs. 10%, p<0.01 [0.935-0.969]). Factors such as age group 40-59 (OR: 1.29, p=0.022), coronary artery disease (OR: 2.181, p<0.01), sepsis (OR: 1.358, p<0.01), and chronic obstructive pulmonary disease (COPD) (OR: 1.238, p<0.01) were identified as individual risk factors affecting the 30-day readmission rate, as presented in Table 2.

**Table 2:** Individual risk factors impacting the 30-day readmission

	Odds ratio (OR) in the presence of readmission	p-value
Age group 40–59	1.29	0.022
Coronary artery disease	2.181	P<0.01
Sepsis	1.358	P<0.01
Chronic obstructive pulmonary disease (COPD)	1.238	P<0.01

In the subsequent phase, the care transition team used the SDOH questionnaire for high-risk groups and contacted 432 patients, 207 (48%) of whom were female. The mean readmission rate post-intervention was 39 (9%) patients. The 60-79 age group had a higher readmission rate compared to the 20-39 and 40-59 age groups (41% vs. 23% and 36%, p<0.01 [0.69-0.90]), as shown in Table 3.

**Table 3:** Thirty-day readmission demographic after the intervention Values are presented as numbers (n)

	Total cases n = 39	Demographic in percentage %
Race		
African American	7	19%
White	9	26%
Asians	2	4%
Hispanic	13	31%
Others	8	20%
Age		
20–39	09	23%
40–59	14	36%
60–79	16	41%

The Hispanic population had a higher readmission rate than other races (31% vs. 19% vs. 26% vs. 4% vs. 20%, p=0.023 [0.78-0.92]), as indicated in Table 3. Age group 60-79 (OR: 1.125, p = 0.012), coronary artery disease (OR: 1.11, p<0.01), sepsis (OR: 2.23, p=0.032), and COPD (OR: 1.1, p<0.01) continued to be individual risk factors for readmission, as shown in Table 4. However, the overall readmission rate declined after implementing a care transition team (18% vs. 9%, p=0.031).

**Table 4:** Individual risk factors impacting the 30-day readmission post-intervention

	Odds ratio (OR)	p-value
Age group 60-79	1.125	p = 0.012
Coronary artery disease	1.11	p < 0.01
Sepsis	2.23	p = 0.032
Chronic obstructive pulmonary disease (COPD)	1.1	p < 0.01

## Discussion

Study highlights

Our two-phase study highlights the vital role of care transition teams in reducing hospital readmission rates and alleviating the financial burden on healthcare institutions. The initial phase established a baseline readmission rate and identified risk factors such as age, race, and specific comorbidities. The second phase demonstrated the positive impact of care transition teams and targeted interventions, resulting in a significant reduction in readmission rates.

The findings of this study underscore the importance of optimizing inpatient care, care transitions, and case management to improve patient outcomes and reduce readmission rates. By implementing care transition strategies consistently and addressing high-risk patients' needs, hospitals can mitigate the adverse effects of readmissions on both patients and healthcare systems.

Furthermore, this study emphasizes the necessity of addressing social determinants of health and providing tailored support during care transitions for specific populations. The care transition team's targeted approach, which utilized the Social Determinants of Health Questionnaire, effectively reduced readmission rates.

Factors contributing to hospital readmissions 

Hospital readmissions can result from various factors, including patients' chronic or multiple medical conditions, which make medical management more complex. The risk of readmission increases with age due to comorbidities, polypharmacy, and more significant post-discharge social care needs [5]. Mental illness also contributes to higher readmission risks.

Inadequate follow-up post-discharge, such as insufficient information handover to general practitioners, patients, and home caregivers, can lead to readmissions. Social factors like limited transportation, financial constraints, and difficulties accessing care play a role, too [6]. Medication non-compliance is a modifiable risk factor, and adherence can help identify patients at higher readmission risk [7]. Demographic characteristics, like patients' residential area, health insurance status, or limited access to healthcare, may cause treatment delays and subsequent readmissions [8].

Hospital readmissions are often considered an indicator of quality medical care [9]. Hospital-related causes include improper treatment, inadequate care transfer from secondary to primary care, and ineffective social assistance plans, especially for older people [10-12]. In addition, higher readmission rates are associated with insufficient post-discharge care and complications arising from incorrect dosages or missing prescriptions [13]. Increased pressure to reduce inpatient lengths of stay also contributes to readmissions [5]. Hospitals with manageable nurse workloads, such as those with higher staffing levels of registered nurses, tend to have lower readmission rates [14,15].

Impact of hospital readmissions

Hospital readmissions pose a significant burden on the healthcare system, resulting in increased costs and strained resources. For example, the Center for Health Information and Analysis (CHIA) estimates that Medicare's annual readmission cost is $26 billion, with $17 billion deemed avoidable [16]. Factors contributing to these heightened costs include the need for additional diagnostic tests and procedures, extended hospital stays, and increased medication use. Moreover, readmitted patients often require further rehabilitation services and home healthcare, which escalates costs [17].

A JAMA 2011 study found that patients readmitted within 30 days of initial discharge reported significantly lower satisfaction with their hospital experience compared to those not readmitted [18]. Another systematic review published in the American Journal of Managed Care revealed that readmissions correlated with lower patient satisfaction scores in various domains, such as communication with healthcare providers and overall hospital experience [19].

Readmitted patients face more extended hospital stays, higher mortality rates, and increased healthcare costs, leading to increased pressure on hospital resources, longer wait times, and a decline in care quality. Consequently, reducing hospital readmissions has become a priority for healthcare providers and policymakers, aiming to alleviate overcrowding and enhance patient outcomes [20].

Strategies to reduce hospital readmissions

Enhanced Discharge Planning, Care Coordination, and Transitional Care Programs

Elderly patients recently discharged from the hospital face the highest risk of readmission [21]. Delays in hospital discharge for this population can result in worsened health outcomes and increased long-term care needs [22]. Inadequate discharge planning and transitional care programs, particularly for elderly patients, can raise readmission rates and negatively impact patients' and caregivers' quality of life [23]. Up to 79% of readmissions are preventable, often due to a lack of coordinated care post-discharge [24].

Transitional care interventions, studied across hospital and community settings, have demonstrated reduced readmission rates when implemented after discharge [21]. These interventions focus on patient and caregiver education and treatment coordination between acute and outpatient settings [25]. Successful transitional care programs decrease readmission rates and significantly reduce healthcare costs [21].

To minimize the risk of adverse outcomes like readmissions, hospitals should prioritize developing effective transitional care programs for elderly patients [23].

Patient Education

A patient-centered approach is crucial when educating patients before hospital discharge [24]. This involves using understandable language and considering cultural factors that may impact healthcare [24]. Evaluating a patient's health literacy, which encompasses reading, writing, and speaking proficiency, is essential for effective communication [24]. In healthcare, this includes the ability to understand hospital instructions and adhere to treatment plans post-discharge. Resources such as printouts, animations, and patient education websites can aid in educating patients with low literacy skills [24].

A study examining education in patients with chronic diseases, specifically congestive heart failure (CHF), demonstrated a significant 35% reduction in rehospitalization and death [26]. This outcome was attributed to increased compliance with outpatient treatment and lifestyle changes among many patients, ultimately leading to decreased death and rehospitalization rates [26].

Technology

Information and communications technology (ICT) in healthcare can aid in managing chronic illnesses effectively [27]. ICT can take various forms, including telemedicine, telehealth, and home telecare [25]. Telemedicine enables physicians to examine patients through technology, while telehealth focuses on patient and provider education [27]. Home telecare, a branch of telemedicine, supports patients in home or community settings [28]. Although the cost-effectiveness of telemedicine in chronic disease management is limited, telecare has shown significant cost reductions in managing CHF [27]. For example, one study reported an 83% decrease in CHF patient admission rates after the intervention [29].

Health information exchange (HIE) facilitates the transfer of electronic health information, such as clinical data, lab test results, and medication lists, among patients, providers, and health organizations [30]. HIE has proven beneficial in sharing critical patient health information between primary care providers and specialists [30]. While only modestly associated with reduced readmission rates, HIE has led to significant cost decreases by eliminating unnecessary duplication of medical exams, lab studies, and imaging [30].

The study's significant limitation lies in its retrospective design, which could introduce various biases and confounding factors. Retrospective studies depend on previously collected data, which may be incomplete or inaccurate, leading to potential errors in the analysis. Researchers have limited control over the quality of their data when relying on historical medical records. As a result, the findings of this study should be approached with caution, given that the retrospective design may compromise the results' accuracy and reliability.

Furthermore, the study did not gather socioeconomic and literacy data, which could be crucial factors influencing the study's outcome. The absence of this information restricts the study's ability to identify and adjust for potential confounding variables, potentially reducing the findings' accuracy and generalizability.

Another limitation is the relatively brief follow-up period during the second phase, which might not offer a comprehensive understanding of the care transition team's interventions' long-term effects. Moreover, conducting the study in a single hospital may limit the generalizability of the findings to other healthcare institutions with different patient populations or practices. Further research involving multiple hospitals and extended follow-up periods would help validate the effectiveness of care transition teams in reducing hospital readmissions.

## Conclusions

Our research demonstrates the significant impact of care transition teams on decreasing readmission rates and reducing financial pressure on healthcare facilities. This can be achieved through improved inpatient care, efficient care transitions, and effective case management, thereby enhancing healthcare outcomes and minimizing mortality rates. The study underlines the importance of early and consistent application of care transition strategies for all patients.

Addressing the specific needs of high-risk patients, including those with particular comorbidities or belonging to a certain age or racial group, is also emphasized. By employing interventions like the Social Determinants of Health Questionnaire, care transition teams can better manage readmission factors. While our study has limitations, it offers valuable insights into the potential benefits of care transition teams. Future research involving larger samples and longer follow-ups will further validate these findings. Our study concludes by stressing the importance of high-quality care and comprehensive case management for sustainable hospital success and financial stability.

## References

[1] Goldfield NI, McCullough EC, Hughes JS, Tang AM, Eastman B, Rawlins LK, Averill RF (2008). Identifying potentially preventable readmissions. Health Care Financ Rev.

[2] Shaw JA, Stiliannoudakis S, Qaiser R, Layman E, Sima A, Ali A (2020). Thirty-day hospital readmissions: a predictor of higher all-cause mortality for up to two years. Cureus.

[3] Pugh J, Penney LS, Noël PH (2021). Evidence based processes to prevent readmissions: more is better, a ten-site observational study. BMC Health Serv Res.

[4] Upadhyay S, Stephenson AL, Smith DG (2019). Readmission rates and their impact on hospital financial performance: a study of Washington hospitals. Inquiry.

[5] Shalchi Z, Saso S, Li HK, Rowlandson E, Tennant RC (2009). Factors influencing hospital readmission rates after acute medical treatment. Clin Med (Lond).

[6] Fluitman KS, van Galen LS, Merten H (2016). Exploring the preventable causes of unplanned readmissions using root cause analysis: Coordination of care is the weakest link. Eur J Intern Med.

[7] Rosen OZ, Fridman R, Rosen BT, Shane R, Pevnick JM (2017). Medication adherence as a predictor of 30-day hospital readmissions. Patient Prefer Adherence.

[8] Kirby SE, Dennis SM, Jayasinghe UW, Harris MF (2010). Patient related factors in frequent readmissions: the influence of condition, access to services and patient choice. BMC Health Serv Res.

[9] Landrum L, Weinrich S (2006). Readmission data for outcomes measurement: identifying and strengthening the empirical base. Qual Manag Health Care.

[10] Witherington EM, Pirzada OM, Avery AJ (2008). Communication gaps and readmissions to hospital for patients aged 75 years and older: observational study. Qual Saf Health Care.

[11] van Walraven C, Seth R, Austin PC, Laupacis A (2002). Effect of discharge summary availability during post-discharge visits on hospital readmission. J Gen Intern Med.

[12] Mistiaen P, Francke AL, Poot E (2007). Interventions aimed at reducing problems in adult patients discharged from hospital to home: a systematic meta-review. BMC Health Serv Res.

[13] Benbassat J, Taragin M (2000). Hospital readmissions as a measure of quality of health care: advantages and limitations. Arch Intern Med.

[14] McHugh MD, Ma C (2013). Hospital nursing and 30-day readmissions among Medicare patients with heart failure, acute myocardial infarction, and pneumonia. Med Care.

[15] Weiss ME, Yakusheva O, Bobay KL (2011). Quality and cost analysis of nurse staffing, discharge preparation, and postdischarge utilization. Health Serv Res.

[16] Bose KS, Sarma RH (1975). Delineation of the intimate details of the backbone conformation of pyridine nucleotide coenzymes in aqueous solution. Biochem Biophys Res Commun.

[17] Jha AK, Orav EJ, Epstein AM (2011). Low-quality, high-cost hospitals, mainly in South, care for sharply higher shares of elderly black, Hispanic, and medicaid patients. Health Aff (Millwood).

[18] Kansagara D, Englander H, Salanitro A, Kagen D, Theobald C, Freeman M, Kripalani S (2011). Risk prediction models for hospital readmission: a systematic review. JAMA.

[19] Boulding W, Glickman SW, Manary MP, Schulman KA, Staelin R (2011). Relationship between patient satisfaction with inpatient care and hospital readmission within 30 days. Am J Manag Care.

[20] Krumholz HM, Lin Z, Keenan PS (2013). Relationship between hospital readmission and mortality rates for patients hospitalized with acute myocardial infarction, heart failure, or pneumonia. JAMA.

[21] Finlayson K, Chang AM, Courtney MD (2018). Transitional care interventions reduce unplanned hospital readmissions in high-risk older adults. BMC Health Serv Res.

[22] Pellett C (2016). Discharge planning: best practice in transitions of care. Br J Community Nurs.

[23] Zurlo A, Zuliani G (2018). Management of care transition and hospital discharge. Aging Clin Exp Res.

[24] Polster D (2015). Preventing readmissions with discharge education. Nurs Manage.

[25] Feltner C, Jones CD, Cené CW (2014). Transitional care interventions to prevent readmissions for persons with heart failure: a systematic review and meta-analysis. Ann Intern Med.

[26] Koelling TM, Johnson ML, Cody RJ, Aaronson KD (2005). Discharge education improves clinical outcomes in patients with chronic heart failure. Circulation.

[27] Celler BG, Lovell NH, Basilakis J (2003). Using information technology to improve the management of chronic disease. Med J Aust.

[28] Kinsella A (1998). Home telecare in the United States. J Telemed Telecare.

[29] Roglieri JL, Futterman R, McDonough KL, Malya G, Karwath KR, Bowman D, Skelly J (1997). Disease management interventions to improve outcomes in congestive heart failure. Am J Manage Care.

[30] Kash BA, Baek J, Davis E, Champagne-Langabeer T, Langabeer JR 2nd (2017). Review of successful hospital readmission reduction strategies and the role of health information exchange. Int J Med Inform.

